# Characteristics and Biological Activities of a Novel Polysaccharide R1 Isolated from *Rubus chingii* Hu

**DOI:** 10.3390/foods13233791

**Published:** 2024-11-25

**Authors:** Zhier Lin, Sisi Liu, Yi Wang, Jianfang Chen, Jihong Huang, Ruqiang Huang

**Affiliations:** 1 School of Life Sciences, Guangdong Provincial Engineering Technology Research Center for Drug and Food Biological Resources Processing and Comprehensive Utilization, South China Normal University, Guangzhou 510631, China; 2023023034@m.scnu.edu.cn (Z.L.); 13265176853@163.com (S.L.); wangy6467@163.com (Y.W.); 13416409794@163.com (J.C.); 2 School of Biological Engineering, Henan University of Technology, Zhengzhou 450001, China; 3 College of Food and Biological Engineering, Henan University of Animal Husbandry and Economy, Zhengzhou 450046, China; 4 State Key Laboratory of Crop Stress Adaptation and Improvement, College of Agriculture, Henan University, Kaifeng 475004, China; 5 Food Laboratory of Zhongyuan, Luohe 462300, China

**Keywords:** *Rubus chingii* Hu, polysaccharide, antioxidant activity, anti-cancer activity

## Abstract

Raspberry (*Rubus chingii* Hu) is a Chinese herb that is rich in nutrients and has anti-inflammatory, antibacterial, antioxidant, anti-allergic, hypoglycemic, and other effects. A water-soluble polysaccharide was extracted from raspberry by using hot water extraction then purified by DEAE-Sepharose Fast Flow column chromatography. The structural characteristics of the polysaccharide (R1) are as follows: the molar ratio of the monosaccharide composition is Ara:Gal:Xyl:Glc:Man = 31.15:27.64:13.61:13.48:10.60; the molecular weight is 32,580 Da; the methylation results show that 5-Araf is the main chain and there is a presence of 3,6-Galp, 4-Xylp, and 2,3,5-Araf branches, and that terminal Araf (T-Araf) is the major telomeric sugar. It contains α and β glycosidic bonds and is highly branched, with the presence of a helical structure. In the in vitro antioxidant assay, R1 showed the highest scavenging of superoxide anion radicals at 70.38%, followed by the scavenging of DPPH radicals at 52.9% and the scavenging of hydroxyl radicals at 29.28%. In immunomodulation and anti-cancer experiments, R1 did not significantly inhibit or promote RAW264.7 cells but was able to increase the expression of anti-inflammatory cytokines in a concentration-dependent manner. It also significantly inhibited cancer cell survival. R1 enhances immunity by limiting the proliferation of cancer cells primarily through direct inhibition while promoting the secretion of pro-inflammatory cytokines. These findings reveal the potential benefits of raspberry polysaccharides and provide evidence for developing immunologically functional products from raspberry polysaccharides.

## 1. Introduction

Raspberry (*Rubus chingii* Hu), a perennial shrub belonging to the Rosaceae family, is extensively found across China, Central Asia, North America, and Europe [1], and current research is mainly focused on raspberry fruits, which have been cultivated for hundreds of years as a nutritious food and herbal medicine. Its fruit value is derived from the high content of healthy compounds such as ellagic acid, sugars, vitamins, minerals, carotenoids, and phenolics (anthocyanins, flavonoids, and phenolic acids) [1,2]. On this basis, it has diverse and effective medicinal effects such as anti-inflammatory, antibacterial, antioxidant, anti-allergic, and hypoglycaemic effects [3,4]. For many years, the unripe dried raspberry fruit has been used by herbalists to treat spermatorrhea, dysuria, impotence and premature ejaculation, and dimming of the eyes [5]. Existing studies have mainly focused on raspberry fruits and less on raspberry leaves. In China, people in some areas use dry raspberry leaves to make tea, and the brewed tea has a sweet flavor and is called sweet tea, which is claimed to have the effect of quenching thirst and clearing fat [6].

Polysaccharides are natural macromolecular polymers widely found in plants [7], animals [8], and microorganisms [9]. Due to the close relationship between structure and function, different methods of extraction and purification may affect the type of polysaccharide and therefore its biological activity [10]. Polysaccharides have various biological activities such as antiviral [11], anti-cancer [12], antioxidant [13], immunomodulatory [14], and hypoglycemic [12] activities. Due to its safety and low side effects, it is considered as an ideal ingredient for food, pharmaceutical, and beauty products [15].

Ten raspberry fruit polysaccharides have been publicly reported. It is mainly found that it has an anti-inflammatory effect [16,17,18,19], can alleviate colitis [20], can regulate the immune response by regulating macrophage activity [21,22,23,24], and can also alleviate oxidative stress-induced damage [21,23,24] such as palmitic acid-induced lipotoxicity and urethane-induced cytotoxicity [25,26]. It has also been found to have antioxidant effects, non-enzymatic glycosylation inhibition, alpha-amylase inhibition, the modulation of intestinal microbial diversity, and anti-cancer activity. There is little research on its anti-tumor activity. Zhang et al. [19] observed that the inhibition of raspberry polysaccharides on the proliferation of breast cancer cell line MCF-7 and liver cancer cell line Bel-7402 was both concentration- and time-dependent. Structurally, galacturonic acid-rich polysaccharides were found to show good anti-inflammatory activity and the arabinose content correlated with immunomodulatory activity.

Limited research has explored the connection between the raspberry polysaccharide structure and its bioactive functions and the relationship between the two remains unclear. In this study, using a 0.1 M NaCl-eluted raspberry purified polysaccharide (R1), The fine structure of the purified polysaccharide was characterized by IR spectra, ion chromatography, hydrogen NMR, methylation, and Congo red determination. Then, the antioxidant, anti-inflammatory, and anti-tumor activities of the polysaccharide were analyzed. This study enriched the types of raspberry polysaccharides and compared the structural functions of the extracted new polysaccharides with the discovered polysaccharides, which provided experimental support for the better development of raspberries.

## 2. Materials and Methods

### 2.1. Materials

Commercially available dried raspberry (*Rubus chingii* Hu, Guangzhou, China) fruits, light brown in color, were used. The dried fruits were crushed and passed through an 80-mesh sieve and stored in a sealed environment at 4 °C.

The chemical reagents and materials used in the experiment are: DEAE-Sepharose Fast Flow Gel (Yuanye Biotechnology Co., Ltd., Shanghai, China), monosaccharide standards (Sigma, Steinheim, Germany). And other chemical reagents are analytically pure.

### 2.2. Extraction and Purification of Polysaccharides

The ratio of dried raspberry powder to ultrapure water was 1:20, heated in a water bath at 80 °C, stirred at 150 rpm, and extracted for 2 h. It was extracted again under the same conditions. After vacuum filtration, the filtrate was mixed and concentrated under reduced pressure at 60 °C. The crude polysaccharide was precipitated by adding 4 times the volume of anhydrous ethanol to the concentrated polysaccharide solution.

The crude polysaccharide solution was mixed with a 10% *w*/*v* trichloroacetic acid (TCA) solution by a volume ratio of 1:1, and the deproteinization step was carried out in an ice bath.

The polysaccharide solution was filtered through a microporous filter membrane and added to a DEAE Fast Flow column, followed by elution with deionized water and 0.1 M NaCl. Then, the 0.1 M NaCl eluate was dialyzed (3000 Da) at 4 °C for 72 h to remove NaCl and small molecular impurities. Purification fractions of the raspberry (R1) were obtained after freeze-drying the dialysate.

### 2.3. Physical and Chemical Property Analysis

A direct observation of the apparent morphology and color of R1 was conducted. The solubility of R1 in organic solvents, such as ethanol, acetone, chloroform, and other organic solvents was studied.

Purity analysis of R1: Determine the total sugar content in R1 by a phenol–sulfuric acid reaction, determine the protein content in R1 by a Comas Blue reaction, detect whether starch is contained in R1 by an iodine–potassium iodide reaction, detect whether phenolics are contained in R1 by a ferric chloride test, detect whether reducing sugar is contained in R1 by Ferring’s reagent test, and detect whether pigments, nucleic acid and other weather pigments, nucleic acids, and other impurities are completely removed by full-wavelength scanning.

### 2.4. Characterization of Polysaccharides

#### 2.4.1. Determination of Molecular Weight

Gel permeation chromatography (GPC) was used to determine the molecular weight and distribution. A total of 100 µL R1 solution was injected into an Agilent column (PL aquagel-OH), with water as the mobile phase, run at 40 °C at a flow rate of 1 mL/min, and detected with an oscillometric detector. Polyethyleneoxide (PEO) mixed with polyethyleneglycol (PEG) standards was used as a reference.

#### 2.4.2. Analysis of Monosaccharide Composition

The Thermo ICS5000 ion chromatography system (ICS5000, Thermo Fisher Scientific, Waltham, MA, USA) equipped with a Dionex^TM^ CarboPac^TM^ PA20 (150 × 3.0 mm, 10 μm) liquid chromatography column (Agilent, Santa Clara, CA, USA) and an electrochemical detector (ECD, Aglient, CA, USA) was used to analyze the monosaccharide fractions. The monosaccharides analyzed included the following: fucose (Fuc), rhamnose (Rha), arabinose (Ara), galactose (Gal), glucose (Glc), xylose (Xyl), mannose (Man), fructose (Fru), ribose (Rib), galacturonic acid (Gal-UA), glucuronic acid (Glc-UA), guluronic acid (Gul-UA), and mannuronic acid (Man-UA).

#### 2.4.3. IR Spectroscopy

The infrared spectra (IR) of R1 samples were obtained using an infrared spectrometer (Nicolet 6700, Thermo Fisher Nicolet, Waltham, MA, USA) in the frequency range of 700–4000 cm^−1^. The R1 sample (1 mg) was mixed with KBr powder, well ground, and pressed into 1 mm particles for FT-IR measurements.

#### 2.4.4. Methylation Analysis

The methylated alditol acetate assay could reveal the glycosidic linkages of the polysaccharide [27]. R1 was methylated by iodomethane and then hydrolyzed using trifluoroacetic acid, followed by reduction with NaBD_4_ and finally acetylation to yield a partially methylated alditol acetate (PMAA) [28]. Next, the collected PMAA was analyzed using GC-MS (7890B, Agilent, Santa Clara, CA, USA) equipped with a DB-5ms capillary column (Agilent 122-5532, USA).

#### 2.4.5. NMR Spectroscopy

A total of 20 mg of R1 samples was fully dissolved in 0.8 mL of D_2_O at room temperature. ^1^H NMR spectra were collected on a Bruker AVANCE NEO spectrometer (Brucker, Karlsruhe, Germany) at 600 MHz.

#### 2.4.6. Congo Red Experiment

According to the method of Tang et al. [29], 1 mL of the R1 solution (2 mg/mL) and an equal volume of 0.1 mmol/L aqueous Congo red solution was placed in a test tube and mixed well, then 2 mL of different concentrations of an aqueous NaOH solution was added and mixed well so that the concentrations of NaOH in the solution were 0, 0.05, 0.1, 0.15, 0.2, 0.25, 0.3, 0.35, and 0.4 mol/L, respectively. The solution was placed for 30 min, and the maximum absorption wavelength was detected by a UV-Vis spectrophotometer (Agilent Cary UV-Vis Compact Peltier, Shanghai, China) at 400–700 nm and measured three times in parallel.

#### 2.4.7. Microstructure Observation

The molecular morphologies of R1 were observed using a scanning electronic microscope (SEM) (FEI Quanta 250 FEG, Fremont, CA, USA). Samples were coated with a thin layer of platinum, placed on a substrate, and the images were observed under high vacuum at 2.0 kV with magnification.

### 2.5. Antioxidant Activities of Polysaccharides

#### 2.5.1. Determination of Hydroxyl Radical Scavenging Capacity

At the beginning of the reaction, R1 samples with different concentration gradients were aspirated, an FeSO_4_ solution and salicylic acid–ethanol solution were added, and finally, a H_2_O_2_ solution was added [30]. After mixing well, the solution was incubated in a constant temperature water bath at 37 °C for 30 min. The absorbance value at 510 nm was determined by enzyme labeling. Ultrapure water was used as a blank control and vitamin C (VC) of the same concentration was used as a positive control. The scavenging rate was used to evaluate the ability of the test substance to scavenge hydroxyl radicals. The higher the value, the better the scavenging activity. The formula for calculating the scavenging rate is as follows:

(1) $$S=\left(1-\frac{A_{X}-A_{X0}}{A_{0}}\right)\times 100\%$$

In the equation, A_0_ is the absorbance value of the blank control; A_X_ is the absorbance value of the sample solution; and A_X0_ is the absorbance value without the addition of a chromogenic agent.

#### 2.5.2. Determination of Superoxide Anion Scavenging Capacity

R1 solutions with different concentration gradients were added to Tris-HCl buffer, and the reaction was carried out at 37 °C for 10 min. Then, an o-benzenetriol solution was added, mixed thoroughly, and left to stand for 4 min at 25 °C before the reaction was rapidly terminated by the addition of 1 drop of a 10 mol/L hydrochloric acid solution, and the absorbance value was measured at 320 nm. VC was the positive control [31]. The scavenging rate was calculated with reference to Equation (1).

#### 2.5.3. DPPH Free Radical Scavenging Capacity

Following reference methods and improvements to Xiang Ruiqi et al.’s [32] study, R1 solutions at different concentrations were added to a DPPH solution, mixed thoroughly and then protected from light at room temperature for 30 min. The absorbance of the solutions at 517 nm was measured. The scavenging rate was calculated with distilled water serving as the blank control and VC at the same concentration as the positive reference. The stronger the antioxidant ability, the more the freer radicals were scavenged and the smaller the absorbance value. The scavenging rate was calculated by referring to Equation (1).

### 2.6. Cellular Activity of Polysaccharides

#### 2.6.1. Cytotoxicity Test

The cytotoxicity of R1 was examined by its effect on RAW264.7 cell survival.

RAW264.7 cells in the logarithmic growth phase were plated and incubated for 24 h. The medium in the original wells was discarded and 100 μL of different concentrations of the R1 sample solution (1, 5, 10, 50, 100, 200, 400, 600 μg/mL) were added to each well, and the control was a medium containing 10% sterile water. The medium was removed after 24 h of incubation in the cell incubator, after which 50 μL of methylene blue staining solution was added to each well, incubated for 1 h, eluted and shaken, and OD_595_ was measured.

Analysis of data (after zeroing): the average value of the control group needed to be derived first and the cell survival rate of each group was derived (experimental data/control average ×100), after which the deviation between each value (deviation between every three wells) was calculated and the toxicity of the sample to the cells was analyzed as a graph.

#### 2.6.2. ELISA Test

The effect of R1 on the expression of IL-6, IL-1β, and TNF-α inflammatory factors in RAW264.7 cells was tested by an ELISA double-antibody sandwich assay. Cells were cultured according to Section 2.6.1 by adding spiked medium (100, 200, 400 μg/mL), a blank control with 10% sterile water, and a positive control with 0.1 μg/mL LPS in 10% sterile water. Pro-inflammatory cytokines were detected with ELISA assay kits.

#### 2.6.3. Anti-Cancer Cell Proliferation Test

The detection of R1 antitumor activity was determined by a CCK-8 assay.

Three cancer cell lines (Hela, HCT116, HepG2) in the logarithmic growth phase were inoculated at 3000 cells/100 μL/well in a 96-well plate and incubated in a cell culture incubator (37 °C, 5% CO_2_) for 24 h. Then, the medium in the original wells was discarded, 100 μL of different concentrations of medium with R1 sample was added, and the control was a medium containing 10% sterile water. After 24 h incubation, 10 μL CCK8 was added to each well, mixed well, incubated in the incubator for 1 h protected from light, and then OD_450_ was measured by enzyme markers.

### 2.7. Statistical Analysis

All presented data were expressed as mean ± standard deviation of three determinations, followed by an analysis of statistical significance using SPSS version 25.0 software. Results for *p* < 0.05 were considered to be statistically significant.

## 3. Results and Discussion

### 3.1. Extraction and Purification

The crude polysaccharide of raspberries obtained by hot water extraction was dehydrated by washing with anhydrous ethanol, acetone, and anhydrous ether and dried, and the yield was 2.09%. The calculated yield was low, which might be related to the degree of dehydration [20]. In the experiments, it was found that the crude polysaccharides precipitated with 76% ethanol were humid and flocculent, dried directly in the oven as a viscous paste, and washed with organic solvents such as anhydrous ethanol, acetone, and ether in a granular form. Therefore, the organic solvents take away a large number of fat-soluble impurities and water, making the yield low.

After purification, the lyophilized polysaccharide yielded 0.06%, was beige cotton wool, extremely soluble in water and hardly soluble in organic solvents.

The total sugar content was measured as 99.68%, determined by the phenol–sulphuric acid colorimetric method. The iodine potassium iodide test, the ferric chloride test, and Felling’s reagent test were all negative, meaning that R1 was free of starch, phenols, and reducing sugars. Protein content was analyzed using the Bradford method, and no protein was measured in R1. A full wavelength scan with no absorption at 520 nm indicated that the pigment had been removed (Figures S1–S3).

### 3.2. Results of Polysaccharide Characterization

#### 3.2.1. Molecular Weight

**Figure 1:**
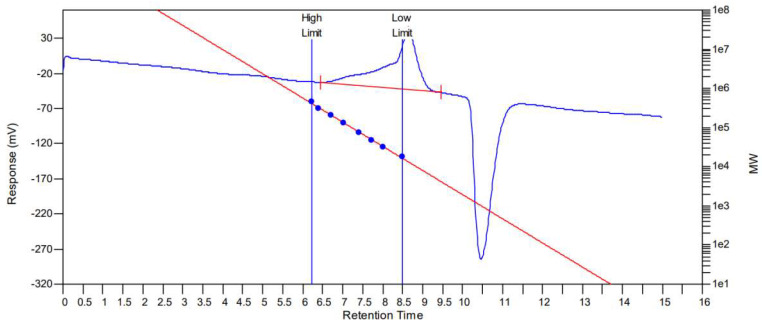
Molecular weight outflow curves of R1.

As presented in Figure 1, results indicated a relatively uniform distribution of R1. There was only one elution peak for R1 in the Gel Permeation Chromatography (GPC), which calculated a MW of 32,580 Da and a Mn of 17,650 Da. The upper part of the peak of the molecular outflow curve showed a symmetrical molecular weight distribution, but with trailing on the high-molecular-weight side, presumably due to intermolecular cross-linking in the salt solution, resulting in a polymer [29]. It is presumed that R1 is a single polysaccharide that can polymerize with each other.

#### 3.2.2. Monosaccharide Composition

**Figure 2:**
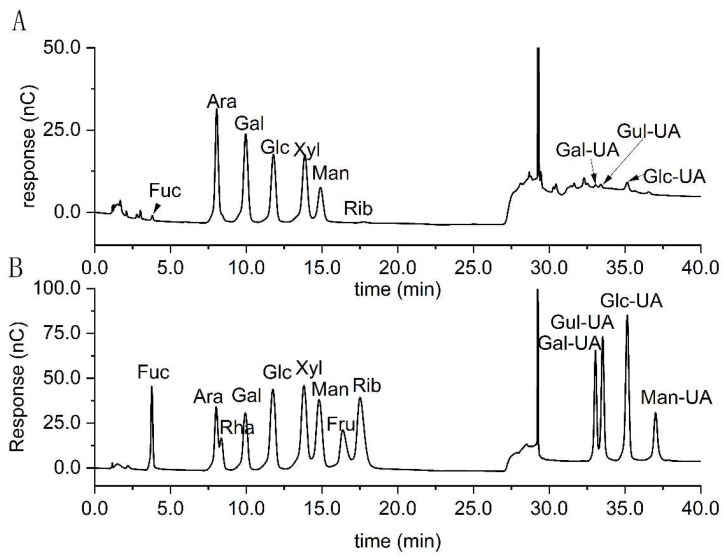
Analysis of monosaccharide composition by ion chromatography. ((**A**) R1; (**B**) standards.).

As showed in Figure 2, standardswere prepared with a mixture of nine monosaccharides and four uronic acids to analyze the monosaccharide composition of R1 based on retention time. The molar ratio of the monosaccharide composition is Ara:Gal:Xyl:Glc:Man:Glc-UA:Fuc:Gul-UA:Gal-UA:Rib = 31.15:27.64:13.61:13.48:10.60:1.34:0.76:0.59:0.54:0.29. It is presumed that arabinose and galactose form the main sugar chain backbone. Moreover, the low uronic acid content (2.47%) indicates that the R1 is a neutral heteropolysaccharide. Compared to the raspberry polysaccharides extracted by other researchers, the R1 extracted in this study are all different in monosaccharide composition, which means that this is a new polysaccharide [18,23,24,25,33,34].

#### 3.2.3. FT-IR Analysis of R1

**Figure 3:**
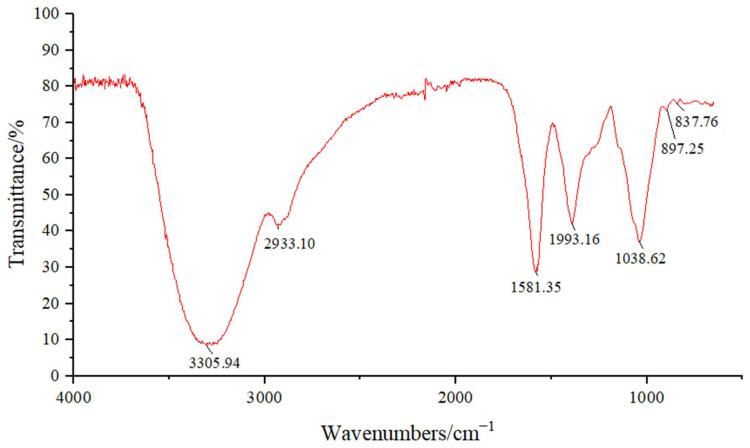
FT-IR spectra of R1.

The absorption peaks characteristic of polysaccharides can be observed in Figure 3, showing a strong, broad peak around 3306 cm^−1^ due to -OH stretching and a weaker peak at 2933.10 cm^−1^ associated with C-H stretching vibrations. The peak at 1581.35 cm^−1^ showed a stretch of the free carboxyl group and a COO symmetry stretch at 1393.16 cm^−1^ [35]. A peak around 1730 cm^−1^ could determine the degree of glyoxylation of glyoxylate-containing polysaccharides, but the absence of a significant absorption peak at the 1730 cm^−1^ attachment indicated that the glyoxylates in the polysaccharides were not esterified [36]. The strong overlapping IR band near 1038.62 cm^−1^ was attributed to C-O stretching vibrations on the pyranose ring [37]. The absorption peaks observed at 897.25 cm^−1^ and 837.76 cm^−1^ suggested the presence of both α- and β-configurations[38].

#### 3.2.4. Methylation Results

The IR spectrogram (Figure S4) of the methylated R1 showed the disappearance of the strong absorption peak (-OH) near 3305 cm^−1^ and the enhancement of the absorption peak caused by the stretching vibration of the methyl group near 2929 cm^−1^ and the deformation vibration at 1385 cm^−1^, indicating successful methylation. By searching the CCRC database and the related literature, the GC-MS results were analyzed. As shown in Table 1, R1 contained 18 different types of glycosidic bonds, with 5-Araf being the main chain and the presence of 3,6-Galp, 4-Xylp, and 2,3,5-Araf branches, as well as terminal Araf(T-Araf) being the main terminal sugar residue. Based on the Hawker equation (DB = (NB + NT)/(NB + NT + NL), where NT, NB, and NL reflect the total percentage of terminal, branched, and linear residues, respectively, the branching degree of R1 was calculated to be 54.76% [39].

#### 3.2.5. NMR

**Figure 4:**
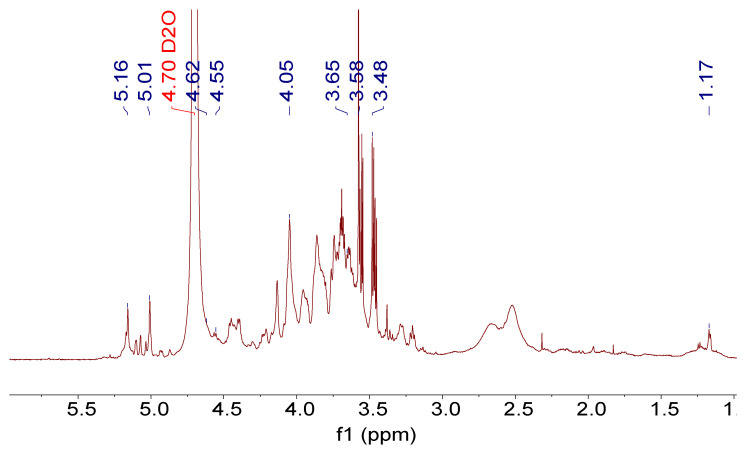
Nuclear magnetic resonance hydrogen spectrogram of R1.

Figure 4 indicates that the NMR hydrogen spectrum showed significant overlap in the range of 3.0–5.5 ppm, a characteristic feature of polysaccharide signals [20]. The strong chemical shift at 4.70 ppm could be attributed to the solvent D_2_O. The end-group hydrogen appeared at 4.5–5.5 ppm, which was indicative of the end-group sugar conformation. The single hydrogen double peak at 5.01 and 5.16 ppm due to proton coupling and the hydrogen shift in the proton on anomeric carbon C1 was greater than 5.0 ppm, indicating that these sugar residues were of the α-type glycosidic bond structure; in addition, the hydrogen shift in the proton on anomeric carbon C1 at 4.62, etc., was less than 5.0 ppm, which identified that R1 also contained the β-type glycosidic bond structure, which was consistent with the IR spectral results. From the peak area, it is inferred that R1 comprises mainly β-type glycosidic bonds with some amount of α-type glycosidic bonds.

In addition, ethanol showed a triple peak at 1.17 ppm (−CH_3_) and a quadruple peak at 3.65 ppm (−CH_2_) in D_2_O but no triple peak at 1.17 ppm in the experimentally obtained hydrogen spectrum, indicating that there was no ethanol residue in the polysaccharide [40].

#### 3.2.6. Congo Red Experiment

**Figure 5:**
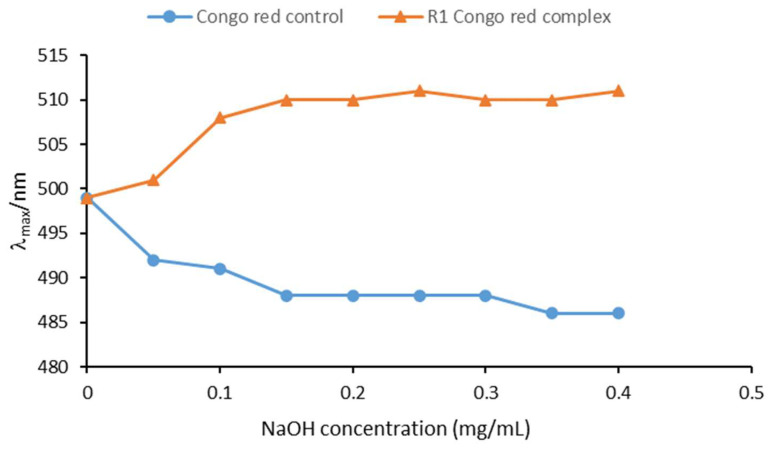
Congo red experiment of R1.

Congo red binds to the helical conformations of polysaccharides such as triple, double, and single helices, resulting in the characteristic red shift of λ_max_ [41]. In Figure 5, it can be seen that the mixing of Congo red with R1 in the absence of sodium hydroxide did not affect the λ_max_ of Congo red. The significant displacement of λ_max_ in low concentrations of sodium hydroxide (0.05 M NaOH) suggested that triple helices may be present in R1 [42]. Moreover, as the alkali concentration increased, λ_max_ showed a rising trend which became relatively weak after stabilization. This phenomenon could be attributed to the transition of the ordered triple helical structure of R1 into a looser helical form or partial disruption under high-alkali-concentration conditions [43,44]. However, the triple helix did not completely unravel to transform into a random coil because Congo red could not bind to randomly curled polysaccharides.

Furthermore, the λ_max_ of R1 remained stable in high concentrations of base solutions (>0.25 M NaOH), similar to the experimental graphs for helical polysaccharides rich in arabinose, galactose, and xylose [23]. It is hypothesized that the Congo red experimental graph line trend correlates with the type of monosaccharide and the way in which the glycosidic bond is attached.

#### 3.2.7. SEM

**Figure 6:**
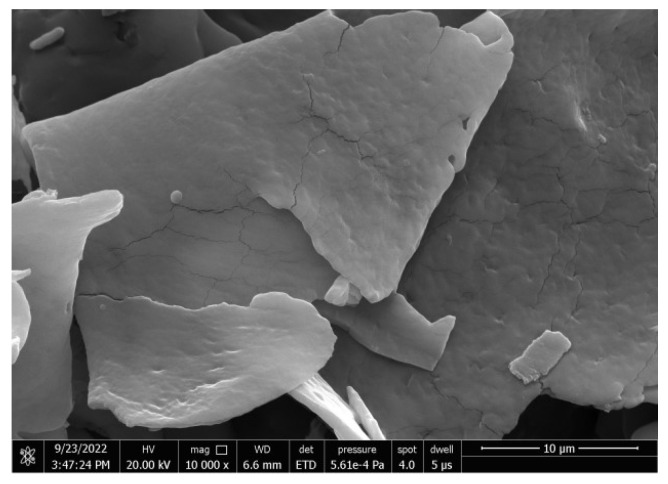
SEM of R1 (10,000×).

Figure 6 shows that R1 contained a large number of irregular lamellar fragments with a relatively smooth surface and dense structure. Some of the polysaccharides may have been degraded during protein removal by the TCA method, forming fragments. At the same time, lower uronic acid content may also result in fewer interaction points between particles, leading to a loose fragmentation state of the polysaccharides [45].

### 3.3. Analysis of Antioxidant Activities of R1

**Figure 7:**
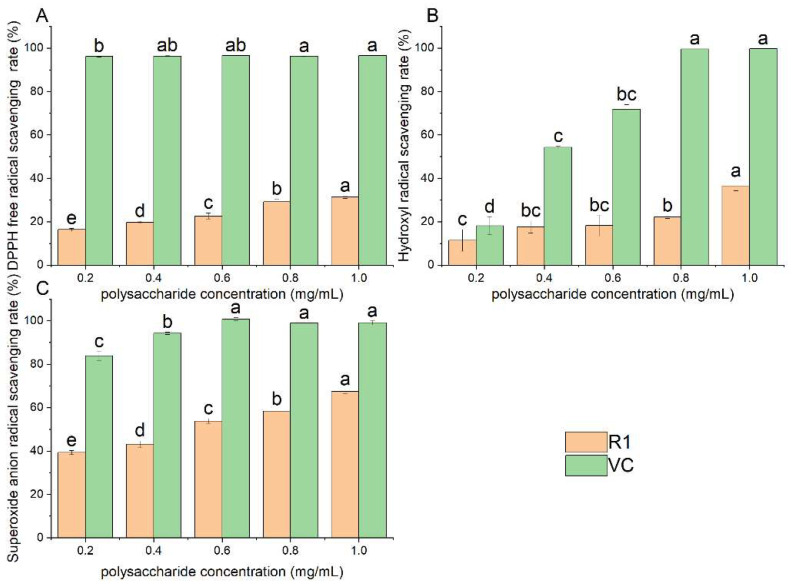
In vitro antioxidant activity of R1: (**A**) DPPH clearance; (**B**) hydroxyl radical scavenging rate; (**C**) superoxide anion radical scavenging rate. Note: different lowercase letters in indicate significant differences, *p* < 0.05.

Figure 7 indicates that the antioxidant capacity of R1 increased with the rise in concentration; however, at the range of 0–1 mg/mL, its antioxidant effect was weaker than that of VC at the same concentration. At the concentration of 1 mg/mL, the scavenging rate of superoxide anion radicals was the highest, at 67.47%, followed by 36.52% for hydroxyl radicals and 31.32% for DPPH.

The structure of polysaccharides determined their biological activity to a certain extent, so the free radical scavenging capacity of polysaccharides was likely to be related to the monosaccharide composition, molecular weight, and degree of branching [30]. The existence form of carboxyl groups was mostly uronic acid, and the content of uronic acid was positively correlated with the antioxidant capacity of polysaccharides and negatively correlated with galactose [46,47]. Compared with the water-soluble raspberry polysaccharide RCP-II extracted by Yu [33], the superoxide anion radical scavenging rate and hydroxyl radical scavenging rate of R1 were slightly lower, while the DPPH scavenging rate was the opposite [20]. The weak antioxidant activity may be related to the low content of uronic acid and high content of galactose in R1. Also, polysaccharides with lower molecular weights had greater antioxidant activity because of the higher number of reducing hydroxyl termini [48]. The relative molecular weight measured in Section 3.2.1 was 32.58 kDa and the branching degree of R1 calculated in Section 3.2.4 was as high as 54.76%, which was considered to have a positive effect on antioxidant activity.

The factors influencing the antioxidant activity of polysaccharides are highly complex, including not only the structure but also the polysaccharide conjugate, the polysaccharide mixture in the crude polysaccharide extract, polysaccharide chelating ions, polysaccharides rich in metal ions, and the chemical modification of polysaccharides [49]. Natural polysaccharides would combine with proteins, peptides, etc., and then provided protons to free radicals lacking electrons to enhance the antioxidant capacity of polysaccharides [50]. When DPPH mixed with an H atom donor, it formed a reduced state and its picrylhydrazyl group (purple-red color) was reduced to picrylhydrazyl hydrazine (light yellow) [51]. The highly branched R1 provided a large amount of hydrogen protons to reduce DPPH, thus improving the DPPH scavenging rate. It may be one of the main ways for raspberry polysaccharides to resist oxidation by providing protons to free radicals to capture the free radicals produced in lipid reactions or by inhibiting the production of free radicals.

### 3.4. ELISA Anti-Inflammatory Assay

**Figure 8:**
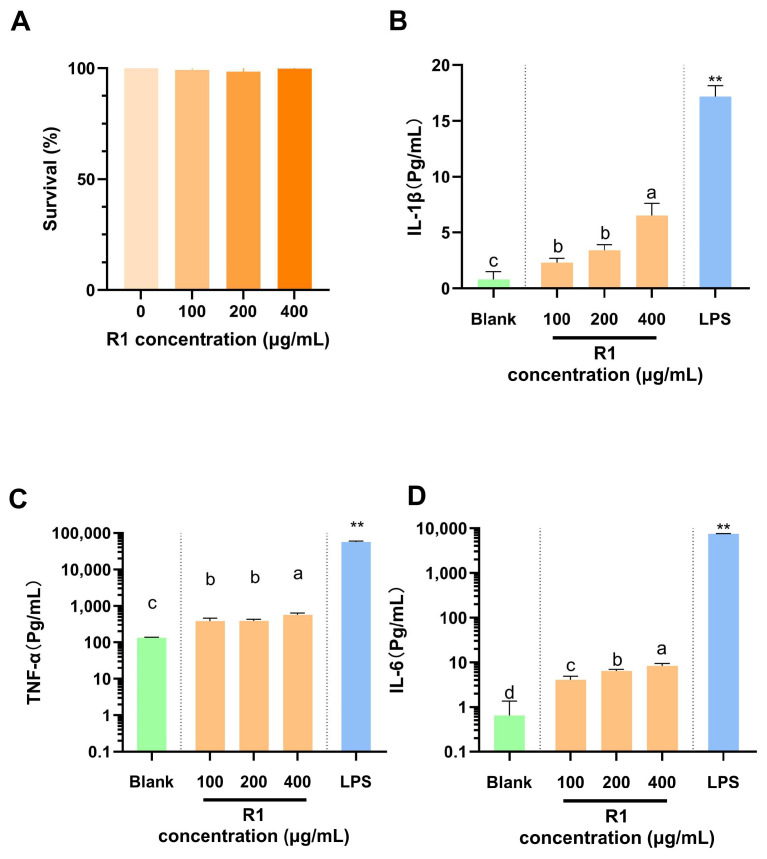
Effects of different concentrations of R1 on RAW 246.7 cell viability and cytokine secretion: (**A**) cytotoxicity; (**B**) IL-1β; (**C**) TNF-α; (**D**) IL-6. Note: different lowercase letters indicate significant differences, *p* < 0.05; ** indicates highly significant differences, *p* < 0.01).

In the range of 0–400 µg/mL, R1 had no significant effect on the apoptosis of RAW264.7 cells, indicating that the polysaccharide had no cytotoxic effect within a certain concentration, but it also had no obvious effect on promoting proliferation (Figure 8A). Although R1 could promote the expression of pro-inflammatory factors IL-6, IL-1β, and TNF-α in macrophages in a dose-dependent manner, only the expression of IL-6 was obvious. High glyoxylate content has been reported to play an important role in the activation of macrophages [52]. Raspberry pulp acidic polysaccharides containing high glyoxylate exhibit significant macrophage activation activity through the increased production of TNF-α, IL-6, and IL-1β [21]. R1 with low uronic acid content can activate macrophages to some extent, but it has no obvious effect on the proliferation of macrophages.

LPS induced macrophages to differentiate into spindle-shaped macrophages, which secreted a variety of pro-inflammatory factors such as IL-6, IL-1β, and TNF-α [53]. Zhang found that raspberry crude polysaccharides at 100 μg/mL inhibited the mRNA expression of IL-6, TNF-α, and iNOS induced by LPS [19]. Also, raspberry pectin polysaccharides were found to reduce colitis by promoting the immune organ index, inhibiting the production of TNF-α and IL-1β. Pectin polysaccharides rich in arabinan side chains could regulate the secretion of IL-6, while galactan tended to regulate IL-10 [17]. Compared with six kinds of pectin polysaccharides and raspberry polysaccharides published now, it was found that raspberry polysaccharides with arabinose content close to 32% had a better anti-inflammatory effect [17]. Therefore, it is speculated that the high content of arabinose in R1 has a great regulatory effect on the expression of pro-inflammatory cytokines, especially IL-6.

M1 macrophages (classically activated macrophages) express a variety of pro-inflammatory mediators, which have strong microbicidal and tumor-killing activities, while M2 macrophages (alternatively activated macrophages) are involved in parasitic infection, tissue remodeling, and tumor progression [54]. M1 and M2 macrophages represent the two extremes of full-spectrum macrophage activation, and they can be transformed in their specific microenvironment to regulate the process of inflammatory diseases [55]. Xu [22] found that raspberry polysaccharides can not only activate macrophages but also regulate the balance between M1 and M2 [22]. The anti-inflammatory effect of R1 may be that it mediates the expression of inflammatory mediators such as cytokines (IL-6, TNF-α) by regulating the phenotype of macrophages, thus regulating immunity and reducing the infiltration of inflammatory cells [56].

### 3.5. Anti-Cancer Activity

Tumors are abnormal growths of cells in the body, which are harmless in benign cases but spread and cause death in malignant cases (cancer). Bioactive compounds with significantly reduced proliferative capacity indicate anti-proliferative or anti-cancer activity. Anti-tumor activity is concentrated in tumor and normal cells, while anti-cancer activity is concentrated exclusively in tumor stem cells [57].

**Figure 9:**
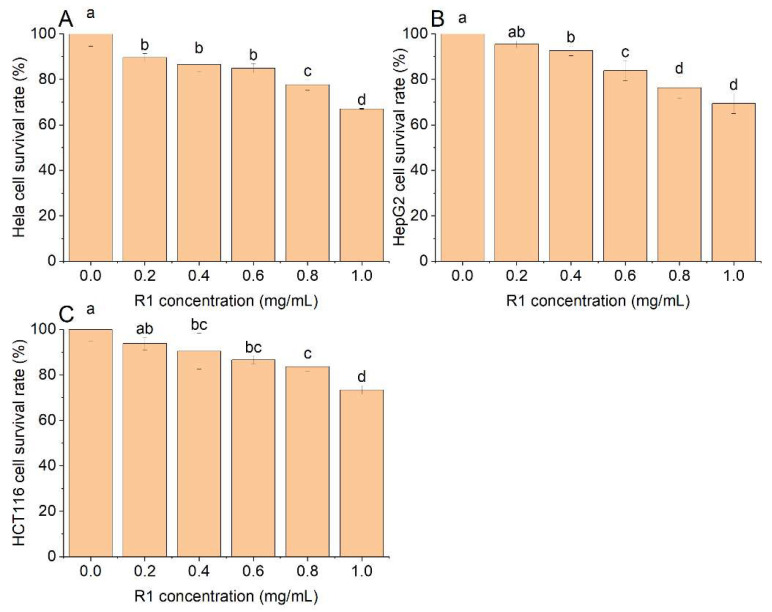
Anti-cancer activity of R1: (**A**) Hela cell survival rate; (**B**) HepG2 cell survival rate; (**C**) HCT116 cell survival rate. Note: lowercase letters indicate significant differences, *p* < 0.05.

Figure 9 indicates that R1 significantly inhibited the growth of human cervical cancer cells (Hela), human liver cancer cells (HepG2), and human colorectal adenocarcinoma cells (HCT116). The result shows that R1 inhibits the proliferation of cancer cells in a positive concentration correlation. When the polysaccharide concentration reached 1 mg/mL, the survival rate of Hela cells after R1 treatment was 67.04%, which was higher than that of HepG2 cells (69.37%) and HCT116 cells (73.36%).

The main way in which tonic polysaccharides exert their anti-tumor effects was by modulating the immune system. Much of the polysaccharide-induced immune enhancement depends on macrophage function, which is the first line of defense of the immune response in the body [58]. Polysaccharides activated effector cells such as macrophages to express cytokines with anti-proliferative activity, such as TNF-α and IL-1β, causing apoptosis and the differentiation of tumor cells [59]. However, R1 did not significantly promote the release of pro-inflammatory factors from macrophages but significantly inhibited cancer cells. Pandya U suggested that polysaccharides derived from mushrooms could induce apoptosis in tumor cells through their direct tumor-suppressive effect [60]. From the above, it can be inferred that the anti-tumor effect of R1 comes partly from the immunomodulation of macrophages and partly from directly killing tumor cells, such as cycle arrest and inducing apoptosis.

## 4. Conclusions

In this study, we reported in detail on a novel water-soluble raspberry heteropolysaccharide, which was rhamnolipid-free and low in glyoxylates (2.47%). It had a relative molecular weight of 32,580 Da and consisted of ten monosaccharides in the molar ratio of Ara:Gal:Xyl:Glc:Man:Glc-UA:Fuc:Gul-UA:Gal-UA:Rib = 31.15:27.64:13.61:13.48:10.60:1.34:0.76:0.59:0.54:0.29. The methylation results showed that 5-Araf was the main chain, 3,6-Galp, 4-Xylp, and 2,3,5-Araf branches were present, and T-Araf was the main terminal sugar residue. Both IR spectra and NMR hydrogen spectra showed that R1 was a typical polysaccharide containing both α and β glycosidic bond conformations. R1 had a good superoxide anion radical scavenging capacity of 67.47% and a scavenging capacity of hydroxyl radicals and DPPH of 36.52% and 31.32%, respectively. However, there was no significant enhancement of macrophage proliferation. R1 significantly promoted the release of the cellular pro-inflammatory factor IL-6 and also increased the release of IL-1β and TNF-α in a concentration-dependent manner. In anti-cancer experiments, R1 was effective in inhibiting the proliferation of cancer cells. Although the main monosaccharide components of polysaccharides were found to have an effect on their biological activity, the exact mechanism remains to be discovered. These findings provide a scientific basis for the further exploration and utilization of raspberry polysaccharides.

## Figures and Tables

**Table 1:** PMAA derivatives for methylation detection and deduced linkages.

Methylation Products	Linkage Pattern	Percentage (%, Area-Based Ratios)
1,4,5-Tri-O-acetyl-1-deuterio-2,3-di-O-methyl-D-arabinitol	5-Araf	17.23
1,4-Di-O-acetyl-1-deuterio-2,3,5-tri-O-methyl-D-arabinitol	T-Araf	16.02
1,2,3,4,5-Penta-O-acetyl-1-deuterio-D-arabinitol	2,3,5-Araf	7.55
1,3,4,5-Tetra-O-acetyl-1-deuterio-2-O-methyl-D-arabinitol	3,5-Araf	5.94
1,2,4,5-Tetra-O-acetyl-1-deuterio-3-O-methyl-D-arabinitol	2,5-Araf	5.6
1,3,4-Tri-O-acetyl-1-deuterio-2,5-di-O-methyl-D-arabinitol	3-Araf	4.47
1,3,5,6-Tetra-O-acetyl-1-deuterio-2,4-di-O-methyl-D-galactitol	3,6-Galp	11.12
1,3,5-Tri-O-acetyl-1-deuterio-2,4,6-tri-O-methyl-D-galactitol	3-Galp	4.83
1,5-Di-O-acetyl-1-deuterio-2,3,4,6-tetra-O-methyl-D-galactitol	T-Galp	2.21
1,5,6-Tri-O-acetyl-1-deuterio-2,3,4-tri-O-methyl-D-galactitol	6-Galp	1.99
1,4,5-Tri-O-acetyl-1-deuterio-2,3-di-O-methyl-D-xylitol	4-Xylp	8.18
1,5-Di-O-acetyl-1-deuterio-2,3,4-tri-O-methyl-D-xylitol	T-Xylp	2.26
1,3,5-Tri-O-acetyl-1-deuterio-2,4,6-tri-O-methyl-D-glucitol	3-Glcp	3.89
1,5-Di-O-acetyl-1-deuterio-2,3,4,6-tetra-O-methyl-D-glucitol	T-Glcp	2.15
1,2,3,5,6-Penta-O-acetyl-1-deuterio-4-O-methyl-D-glucitol	2,3,6-Glc	0.46
1,4,5-Tri-O-acetyl-2-(acetylmethylamino)-2-deoxy-1-deuterio-3,6-di-O-methyl-D-mannitol	4-Manp	2.88
1,2,5-Tri-O-acetyl-1-deuterio-3,4,6-tri-O-methyl-D-mannitol	2-Manp	1.77
1,5-Di-O-acetyl-1-deuterio-2,3,4,6-tetra-O-methyl-D-mannitol	T-Manp	1.44

## Data Availability

The original contributions presented in the study are included in the article/Supplementary Material, further inquiries can be directed to the corresponding authors.

## Supplementary Materials

The following supporting information can be downloaded at: https://www.mdpi.com/article/10.3390/foods13233791/s1, Figure S1: Glucose standard curve; Figure S2: Glucose standard curve; Figure S3: R1 full-wavelength scan; Figure S4: Infrared spectra of methylated R1.
