# Supplementary material for: Characteristics and Biological Activities of a Novel Polysaccharide R1 Isolated from Rubus chingii Hu
Source: Foods. 2024 Nov 25;13(23):3791. doi: 10.3390/foods13233791 (PMC11640516; doi:10.3390/foods13233791)
Supplement: Supplementary file 1 [file foods-13-03791-s001.zip › foods-3286543-supplementary.pdf]

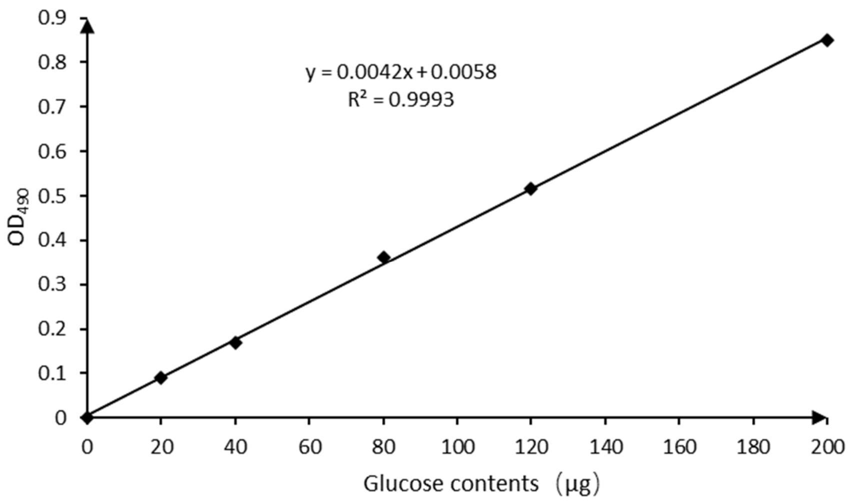

Figure S1. Glucose standard curve

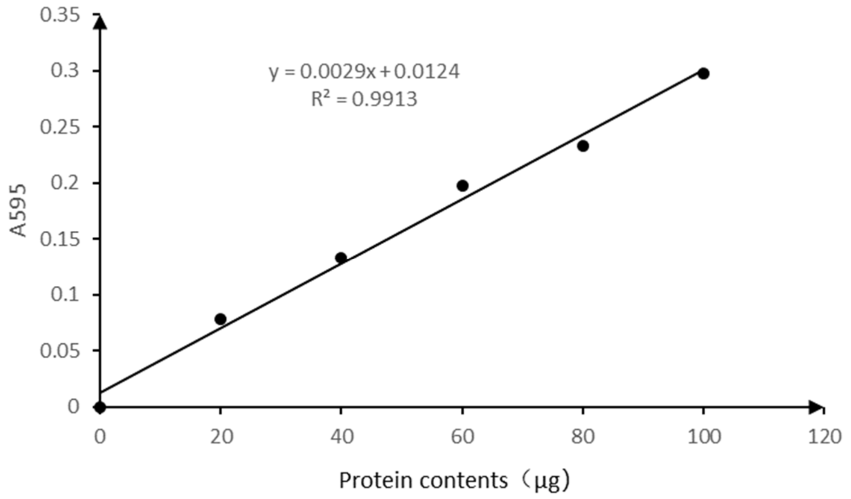

Figure S2. Protein standard curve

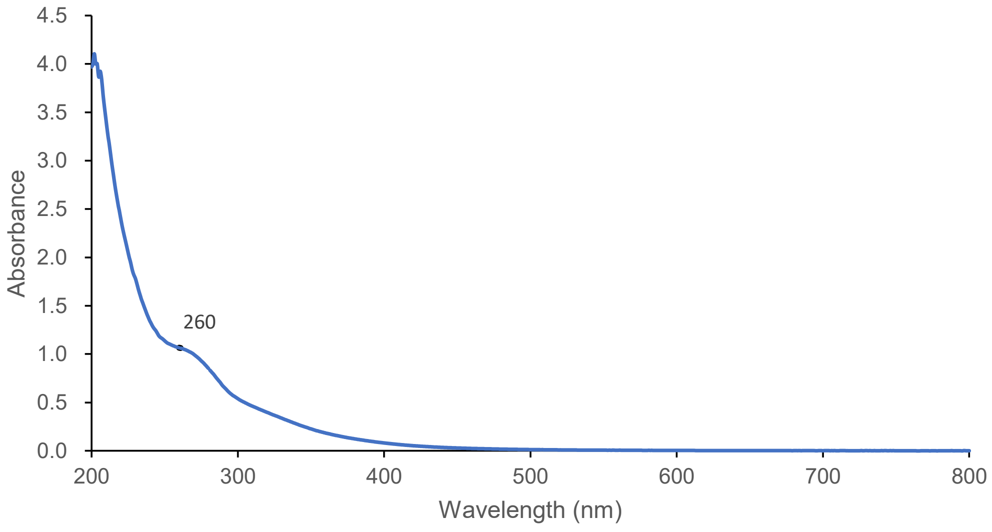

Figure S3. R1 full-wavelength scan

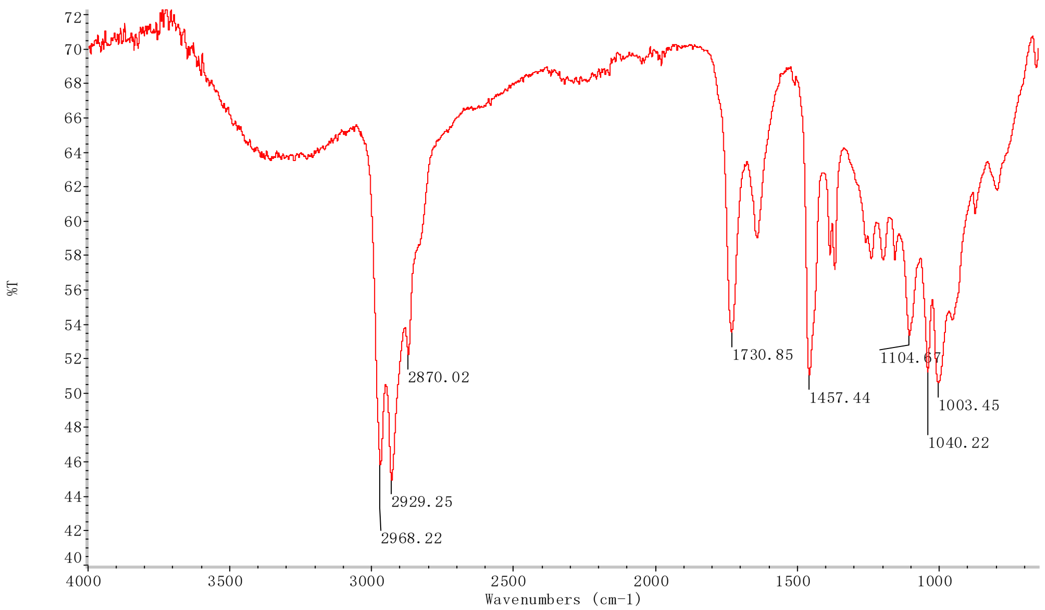

Figure S4. Infrared spectra of methylated R1
